# *KRAS* mutation and primary tumor location do not affect efficacy of bevacizumab-containing chemotherapy in stagae IV colorectal cancer patients

**DOI:** 10.1038/s41598-017-14669-2

**Published:** 2017-10-30

**Authors:** De-cong Sun, Yan Shi, Yan-rong Wang, Yao Lv, Huan Yan, Hui Mao, Zhi-kuan Wang, Zhi-yong Wu, Wei-wei Shi, Guang-hai Dai

**Affiliations:** The Second Department of Oncology, The Chinese PLA General Hospital, Beijing, 100853 China

## Abstract

This study aims to investigate the efficacy of bevacizumab-combined chemotherapy (BCC) in Chinese stage IV colorectal cancer (CRC), and analyze the relationship between clinicopathological features with survival. Patients with stage IV CRC treated with BCC were analyzed retrospectively. 217 metastatic CRC (mCRC) patients were collected, out of which79 were right-sided CRCs and 138 were left-sided ones. Patients with Eastern Cooperative Oncology Group (ECOG) performance status ≤2, single agent chemotherapy, poor/mucous/signet ring cell component, second-and further-line of bevacizumab administration, multiple metastasis sites had comparatively worse survival. Among 141 patients with known *KRAS* status, 55 patients harbored *KRAS* mutation and 86 had wild type *KRAS*. The ORR and DCR were 41.9% and 78.9%, respectively, in patients with wild type *KRAS*, while ORR and DCR was 38.7% and 77.9%, respectively, in patients with *KRAS* mutation. The median PFS of patients with wild type and mutant *KRAS* were 8.38, and9.59 months, respectively; whereas the OS was 23.00 and 21.26 months, respectively for mCRC patients with wild-type and mutant *KRAS*. Cumulatively, our study indicated that BCC was effective and beneficial for Chinese stage IV CRC patients. *KRAS* mutation status and tumor location were not a prognostic factor for survival.

## Introduction

Colorectal cancer (CRC) is one of the most common malignancies globally and is the third major cause of cancer morbidity and mortality^1^. In China, stage IV constitutes more than 10% of the total CRC patients. In stage IV CRC patients, multiple treatment modalities including surgical resection, chemotherapy and radiotherapy are the current methods of choice. In recent years, with the development of our understanding the signaling pathway and mechanism of tumorigenesis, targeted therapy pervaded in anti-cancer regimens and demonstrated further clinical benefits in combination with systemic chemotherapy. Bevacizumab is one of the most successful biologic agents in treating stage IV CRC^2^.

Neoangiogenesis is one of the hallmarks of cancer^3^. One of the important regulators of neoangiogenesis is vascular endothelial growth factor (VEGF)^4^. VEGF expression is highly induced in tumor tissue and is associated with tumor cell proliferation, invasion and metastasis. By downregulating VEGF expression, tumor growth can be inhibited^5^. Bevacizumab, a recombinant humanized monoclonal IgG antibody, selectively combines with VEGF-A inhibiting its binding the VEGF receptors, thus avoiding VEGF-mediated angiogenesis^6,7^. The AVF2107g clinical trial demonstrated that bevacizumab combined with chemotherapy prolonged the survival of patients with stage IV CRC^8^. Based on results from this study, bevacizumab was first approved by the United States Food and Drug Administration (FDA) for stage IV CRC in combination with other cytotoxic agents. Based on numerous clinical trials^9–14^, in the National Comprehensive Cancer Network Clinical Practice Guidelines in Oncology (NCCN Guidelines) for colon and rectal cancer, bevacizumab is recommended as first-, second- and even cross line treatment after first disease progression for stage IV CRC.

While the benefits of treatment with bevacizumab are well studied in American and European patients with stage IV CRC, the effect and safety of treatment with bevacizumab combined chemotherapy in Chinese patients, and whether the *KRAS* mutation status and primary tumor site could affect the prognosis of Chinese stage IV CRC have not been demonstrated clearly. This retrospective study aimed at investigating the efficacy and safety profile of combination treatment with bevacizumab in Chinese stage IV CRC patients and analyzing prognostic factors for predicting patients’ survival.

## Methods

All procedures performed in studies involving human participants were in accordance with the ethical standards of the institutional and/or national research committee and with the 1964 Helsinki declaration and its later amendments or comparable ethical standards. The study protocol was approved by the Institutional Review Board of Chinese PLA General Hospital.

### Study population

This retrospective study included 217 patients with stage IV CRC who had been treated with bevacizumab-containing chemotherapy between May 1, 2011 and August 1, 2015 in Chinese PLA General Hospital. Patients who met the following criterions were included in this study: (1) histologically confirmed colorectal adenocarcinoma with clinical and/or histological evidences of distant metastasis cancer; (2) ECOG performance status (PS) ≤2; (3) life expectancy >3 months; (4) measurable disease consistent with the Response Evaluation Criteria in Solid Tumors (RECIST) version 1.1, (5) adequate organ function, including liver and kidney (total bilirubin ≤1.5-times the institutional upper normal limit, aspartate aminotransferase and alanine aminotransferase ≤2.5-times the institutional upper normal limit, and serum creatinine ≤institutional upper normal limit or creatinine clearance (CCr, calculated using the Cockcroft-Gault formula) ≥50 ml/min); adequate bone marrow function (leucocyte count ≥3000/mm3,neutrophil count ≥1500/mm^3^, platelet count ≥100,000/mm^3^, and haemoglobin ≥9.0 g/dl); and, (6) provided signed informed consent. The key exclusion criteria were as follows: no pathological diagnosis; history of malignancy other than CRC; less than 4 cycles of bevacizumab-containing chemotherapy, thus the tumor response could be evaluated at least once; the presence of clinically significant cardiovascular disease; uncontrolled hypertension; bleeding diathesis or coagulopathy; central nervous system metastasis; use of full-dose anticoagulants or thrombolytics; pregnancy or lactation; non-healing wounds; inability to take therapy on time. Patients with no completed clinicopathological and survival data were also excluded.

### Treatment

All of the 217 patients included were treated intravenously with 5 mg/kg bevacizumab (Avastin; Genentech, San Francisco, CA, USA) every 2 weeks or 7.5 mg/kg every 3 weeks according to different chemotherapy regimens, prior to the chemotherapy. Bevacizumab was administered initially over 90 minutes, and if the first infusion was well tolerated, the second was delivered no less than 60 minutes, and if well tolerated, the subsequent administration was over 30 minutes. Bevacizumab was temporarily or permanently reduced or forbidden in case of serious bevacizumab-related toxicity. Among the 217 patients included in this retrospective study, 75 patients received bevacizumab combined with XELOX chemotherapy, 41 patients received FOLFOX chemotherapy, 67 patients received FOLFIRI chemotherapy; 10 patients received oxaliplatin only; 8 patients received irinotecan alone; 10 received raltitrexed chemotherapy; 6 patients received 5-fluoropyrimidine or capecitabine chemotherapy. The chemotherapy regimens are detailed in Table 1.

**Table 1:** Characteristics and survival analysis of 217 stage IV CRC patients treated with bevacizumab.

**Factor**	**Case (n)**	**Median PFS** (**months**)	**P-value**	**Median OS (months)**	**P-value**
**Gender**			**0.724**		**0.542**
*Male*	120	10.05		19.63	
*Female*	97	10.32		21.07	
**Age**			**0.248**		**0.986**
<*58*	109	9.33		18.96	
≧*58*	108	10.15		21.03	
**ECOG**			0.001*		0.001*
*0–1*	169	10.42		21.00	
*2*	48	6.34		13.96	
**Tumor location**			**0.331**		**0.267**
*Left-sided CRC*	138	10.12		21.26	
*Right-sided CRC*	79	8.58		17.81	
**Differentiation**			**0.277**		0.017*
*High/moderate*	134	10.15		22.95	
*Poor/mucinous/signet ring cell*	83	8.28		16.66	
**No. of metastatic sites**			0.008*		0.025*
*1–2*	156	10.58		21.73	
≥*3*	61	8.15		14.36	
**Line No. of bevacizumab**			0.002*		0.002*
*1*	132	11.40		23.95	
≥*2*	85	6.28		15.61	
***KRAS*** **status (N = 141)**			**0.557**		**0.740**
*Wild Type*	86	8.38		23.00	
*Mutation*	55	9.59		21.26	
**Chemotherapy regimens**					
*Irinotecan-based*	70	11.23	**0.065** ^**a**^	21.75	**0.982** ^**a**^
*Oxaliplatin-based*	113	10.12	0.028* ^**b**^	21.03	0.020* ^**b**^
*Single agent*	34	5.65	0.018* ^**c**^	15.28	0.035* ^**c**^

^*^P < 0.05;

^a^Irinotecan-based combined chemotherapy vs oxaliplatin-based combined chemotherapy;

^b^Oxaliplatin-base combined chemotherapy vs single agent;

^c^Single agent vs irinotecan-based combined chemotherapy.

PFS: progression-free survival; OS: overall survival.

### Clinical Outcome Assessments

The long-term effectiveness measures included PFS, which is defined as the duration from the start of the initial bevacizumab-containing therapy to the first recorded occurrence of disease progression or death; overall survival (OS), which is calculated as the duration from the initiation of the bevacizumab-containing therapy to death or censoring. Patients without an event who still remained in follow-up were censored on the last follow-up date, July 31, 2016. Short-term effective objectives included overall response rate (ORR) and disease-control rate (DCR). Baseline tumor statuses of targeted lesions were evaluated using computer tomography (CT) scan of the chest, abdominal and pelvis. Tumor responses were evaluated at the completion of each 6-week cycle according to RECIST.

### Statistical analyses

For survival analyses, the Kaplan–Meier method was used to estimate the correlation between PFS, OS rates and clinicopathological variables, at 95% CI. The log-rank test was used to compare survival curves. All the statistical analysis was conducted by SPSS 19.0 software package and a *P* < 0.05 was considered as statistically significant.

## Results

### Patient characteristics

217 stage IV CRC patients (120 men, 97 women, median age 58 years old) treated with bevacizumab-containing chemotherapy between May 1, 2011 and August 1, 2015 in Chinese PLA General Hospital were collected and retrospectively analyzed. Baseline demographics and clinical characteristics are summarized in Table 1. 169 patients had an ECOG PS scored 0–1 and 48 patients scored 2 at the initial bevacizumab administration. The number of metastasis in no more than 2 organs was discovered in 156 patients till the last follow-up date. The most common metastatic organ was liver (158 patients), 106 of whom suffered from synchronous liver metastases. One hundred fifty three patients received primary tumor surgery.

### Short-term effect

Among 217 patients, 193 patients had progressive disease, out of whom 118 patients expired by the last follow-up date. The total ORR was 38.3% and DCR was 87.1%. The ORR and DCR were 51.5% and 96.2% when bevacizumab was administered in first-line therapy and 25.5% and 78.2% when administered in second-line therapy. However, ORR and DCR were 10.0% and 53.3% when bevacizumab was given just in third- and forth-line, respectively (Table 2).

**Table 2:** Short-term effect in 217 stage IV CRC patients treated with bevacizumab.

	**N**	**CR (%)**	**PR (%)**	**SD (%)**	**PD (%)**	**ORR**	**DCR**
1^st^ line	132	3(0.7)	67 (50.8)	59 (44.7)	5 (3.8)	51.5%	96.2%
2^nd^ line	55	0	14 (25.5)	29 (52.7)	12 (21.8)	25.5%	78.2%
3^rd^ line	24	0	3 (12.5)	11 (45.8)	9 (37.5)	12.5%	58.3%
4^th^ line	6	0	0	2 (33.3)	4 (66.7)	0%	33.3%
Overall	217	3 (1.4)	80 (36.9)	106 (48.8)	28 (12.9)	38.3%	87.1%

### Long-term effect

**Figure 1:**
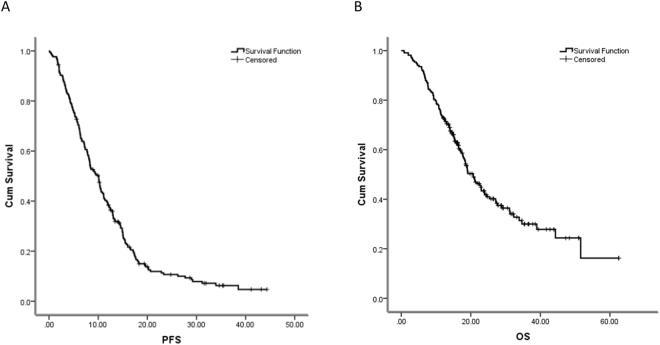
Progression-free survival (PFS) (**A**) and overall survival (OS) (**B**) of 217 stage IV CRC treated with bevacizumab-containing chemotherapy. For details of chemotherapeutic regimen please refer to Table 1. With the median follow-up duration of 27.30 months (range, 11.99–62.52 months), the median PFS and OS were 10.05 and 20.67 months, respectively.

**Figure 2:**
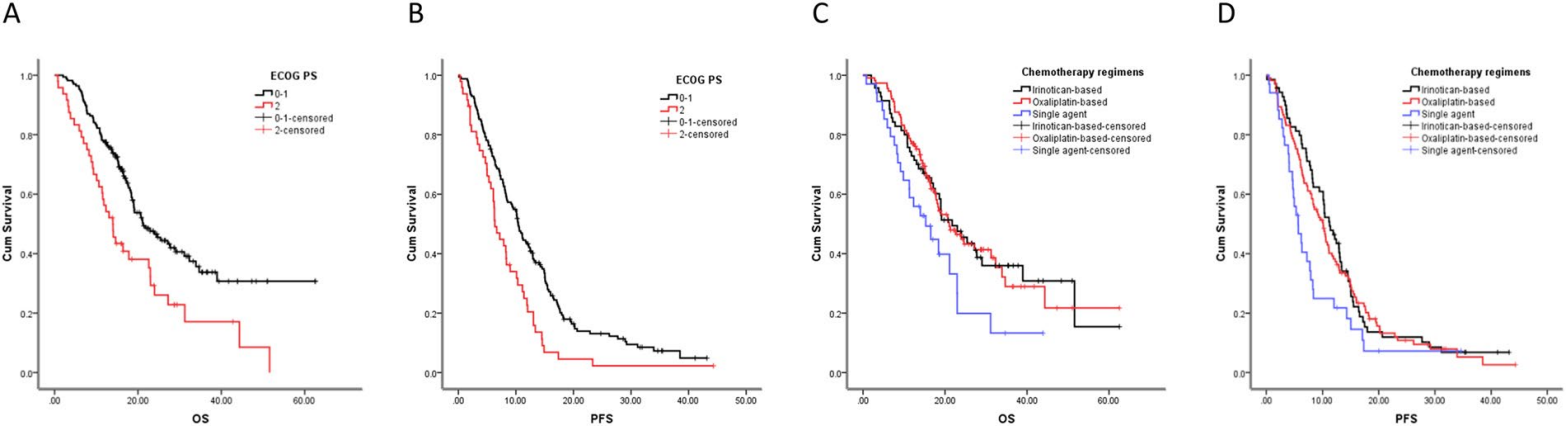
OS (**A**,**C**) and PFS (**B**,**D**) of stage IV CRC patients with different ECOG (**A**,**B**) and under different chemotherapeutic treatment regimens (**C**,**D**). Stage IV CRC patients with ECOG = 2 had significantly worse OS and PFS than patients with ECOG 0–1 (*P* < 0.05) (**A**,**B**). The differences of PFS and OS between oxaliplatin-based combined chemotherapies and irinotecan-based ones were not significant (*P* > 0.05) (**C**,**D**).

With the median follow-up duration of 27.30 months (range, 11.99–62.52 months), the median PFS and OS were 10.05 and 20.67 months, respectively (Fig. 1, Table 1). Stage IV CRC patients with ECOG 2 had worse PFS and OS than patients with ECOG 0–1 (*P* < 0.05) (Fig. 2A,B; Table 1).

As for chemotherapy regimens, single agent such as oxaliplatin, irinotecan, 5-fluoropyrimidine, capecitabine and raltitrexed did not prolong the patients’ survival compared with combinatorial chemotherapeutic regimens. There was no statistically significant difference of PFS and OS between oxaliplatin-based combined chemotherapies and irinotecan-based ones (Fig. 2C,D; Table 1).

**Figure 3:**
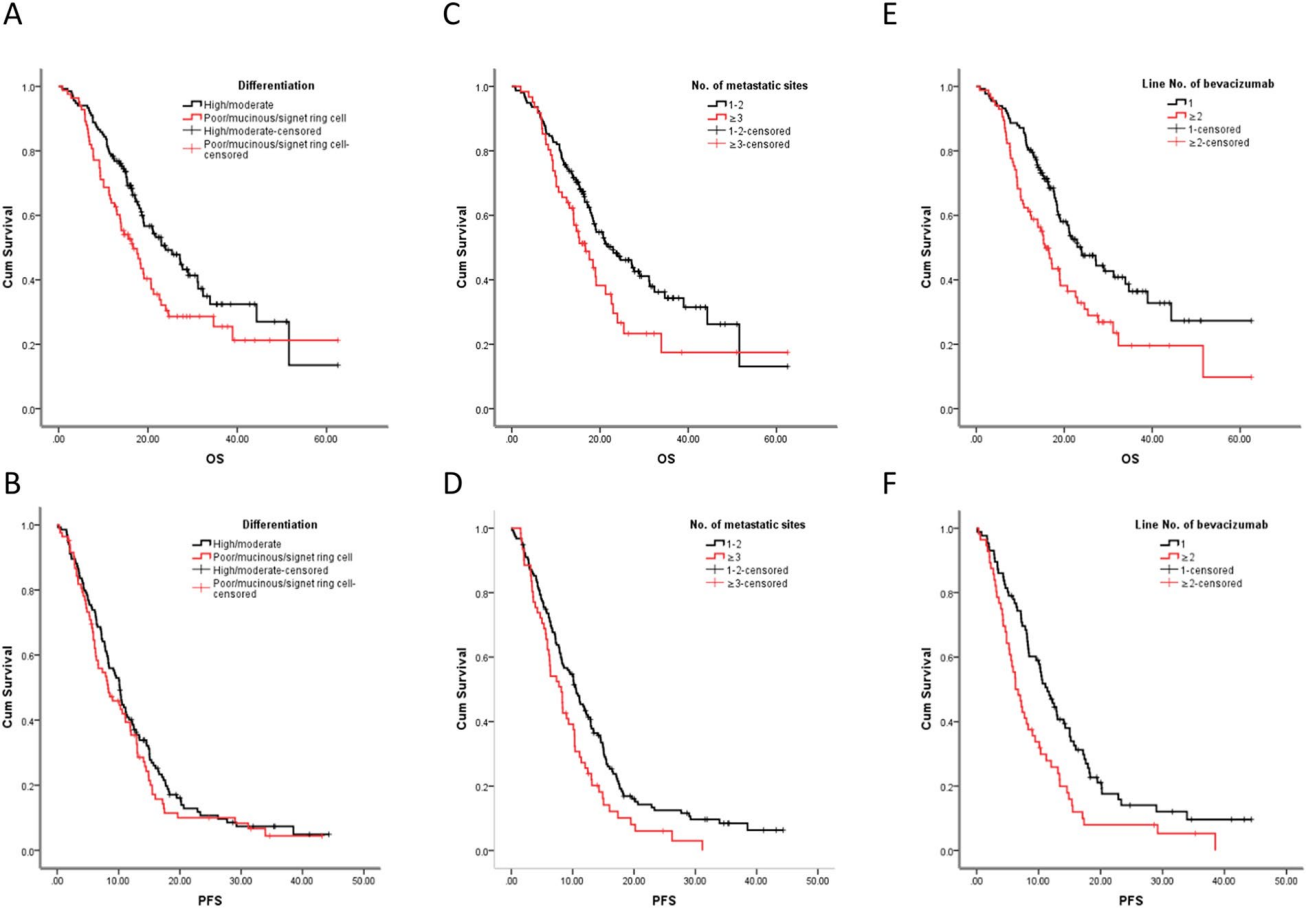
Effect of bevacizumab on OS and progression-free survival of stage IV CRC patients with different differentiation states (**A**,**B**), number of metastatic sites (metastatic sites ≥3 VS 1–2; (**C**,**D**)), and based on line of therapy (first line VS ≥ 2 line; (**E**,**F**)).

Poor/mucous/signet ring cell component had different effort on PFS and OS. Poor differentiation, resembling advanced metastatic stage, significantly shortened the OS (*P* < 0.05) (Fig. 3A) instead of PFS (*P* > 0.05) (Fig. 3B; Table 1). Similar observations were made for number of metastatic sites with patients with more than 2 sites having a significantly lower OS and PFS (*P* < 0.05 in each case) (Fig. 3C,D; Table 1).

We divided time of bevacizumab regimen to two groups – first line and ≥2 (Table 1). The PFS and OS were directly dependent on how quickly bevacizumab was administered (*P* < 0.05 in each case) (Fig. 3E,F; Table 1). The other clinicopathological features did not present differential long-term effect.

### Primary tumor location and KRAS mutation status

**Figure 4:**
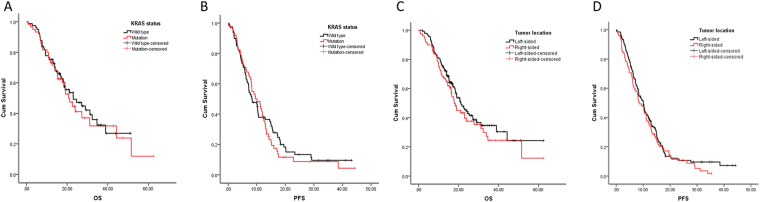
Tumor location and *KRAS* mutation status had no significant effect on PFS and OS of stage IV CRC receiving bevacizumab chemotherapy. OS (**A**,**C**) and PFS (**B**,**D**) of **stage IV** CRC patients with right- or left-sided disease (**A**,**B**) and wild type or mutant of *KRAS* (**C**,**D**) (*P* > 0.05 in each case).

Current evidence suggest that right-sided and left-sided CRC are different diseases^15–17^, and referring to stage IV CRC, right-sided ones had significantly worse survival than left-sided ones^15^. We were interested in the effect of primary tumor location on bevacizumab-related survival. Among 217 enrolled patients, 79 were right-sided CRCs and 138 were left-sided ones. Left- and right-sided CRC had no difference in overall survival following treatment with bevacizumab-containing chemotherapy (Fig. 4A,B; Tables 2,3). Furthermore we analyzed the differences of survival of right- and left-sided CRC in every line of bevacizumab, and there were still no significant differences (*data not shown*).

**Table 3:** Differential effect of bevacizumab on 141 stage IV CRC patients with wild type or mutant *KRAS* status and tumor location.

		**N**	**CR (%)**	**PR (%)**	**SD (%)**	**PD (%)**	**ORR**	**DCR**
***KRAS*** **status (n = 141)**		141	2	55	65	19	40.4%	86.5%
	*Wild*	86	1	35	39	11	41.9%	87.2%
	*Mutation*	55	1	20	26	8	38.2%	85.5%
**Tumor location**		217	3	80	106	28	38.3%	87.1%
	*Left-sided*	138	2	51	68	17	38.4%	87.7%
	*Right-sided*	79	1	29	38	11	37.9%	86.1%

The other commonly used monoclonal antibody in mCRC is cetuximab, which is an anti-EGFR monoclonal antibody^18^. However, *KRAS* mutation is a negative predictive marker for anti-EGFR treatment. For this reason, only mCRC with wild type *KRAS* could be treated with cetuximab^19^. We wondered if *KRAS* mutation status would affect bevacizumab efficacy, so patients who had the *KRAS* status information (n = 141) were divided in to *KRAS* mutation group (n = 55) and wild type group (n = 86). The ORR and DCR were 41.9% and 87.2% in patients with wild type *KRAS*, while 38.2% and 85.5% in patients with mutant *KRAS*. The median PFS and OS of *KRAS* wild type and mutation type were 8.44 vs 9.43 months and 24.61 vs 19.09 months, but the differences were not significant (P > 0.5) (Fig. 4C,D; Tables 1,2).

## Discussion

After bevacizumab was initially approved by the FDA for stage IV colorectal cancer in 2004 based on the results of the AVF2107g RCT, it became one of the standards for first-line, second-line and cross-line therapeutic regimen^20^. The addition of bevacizumab to chemotherapy had been shown to prolong stage IV CRC patients’ survival in multiple randomized controlled clinical trial (RCT) from European and North American countries, where people were mostly Caucasian. A multiple-centered phase III RCT launched in the United States of America in 2004 containing 402 mCRC patients to receive irinotecan, bolus fluorouracil, and leucovorin (IFL) plus bevacizumab and 411 to receive IFL and placebo. The median OS and PFS of combination group were 20.3 and 10.6 months, the ORR was 44.8%. While in IFL group, OS and PFS were 15.6 and 6.2 month, with an ORR of 34.8%^8^. Follow-up clinical trials were conducted to explore the effect of bevacizumab administration during every phase of therapy (important phase III ones are summarized in Table 4).

**Table 4:** Summary of Phase III study with bevacizumab.

**Study**	**Line**	**Cases**	**Group**	**Median PFS (months)**	**Median OS (months)**	**Group**	**Median PFS (months)**	**Median OS (months)**
*AVF2107g*	First	813	B + IFL	15.6	20.3	IFL	6.2	15.6
*BEAT*	First	1989	B + FOLFIRI/FOLFOX/XELOX	11–12	23–26	B + monotherapy	8.6	18.0
*BRiTE*	First			9.9	23.5			
*ARTIST*	First	214	B + mIFL	8.3	18.7	mIFL	4.2	13.4
*N016966*	First	1401	B + XELOX/FOLFOX	9.4	21.3	XELOX/FOLFOX	8.0	19.9
*CARIO3*	Maintenance	558	B + capecitabine	11.7	nd	observation	8.5	nd
*MACRO*	maintenance	480	B + XELOX	10.4	23.2	B	9.7	20.0
*E3200*	Second	829	B + FOLFOX	7.3	12.9	FOLFOX	4.7	10.8
*BEBYP*	Cross	184	B + FOLFIRI/FOLFOX	6.8	14.1	FOLFIRI/FOLFOX	5.0	15.5
*ML18147*	Cross	820	B + oxaliplatin-/irinotecan-based chemotherapy	5.7	11.2	oxaliplatin-/irinotecan-based chemotherapy	4.1	9.8
Current study	First to multiple	217	B + oxaliplatin-/irinotecan-based chemotherapy	10.12/11.23	21.03/21.75	B + single agent chemotherapy	5.65	15.28

nd: no data; B: bevacizumab.

The BEAT study established the combination chemotherapy plus bevacizumab other than monotherapy as the standard therapy for mCRC first-line treatment^11^. However, all RCTs did not get equivalent results. In the N016966 study, even though bevacizumab administration resulted in longer PFS, it did not have the same effect on OS^12^. The CARIO3 and MACRO phase III studies proved the benefit bevacizumab when used only as maintenance therapy^21,22^. The E3200 study, where mCRC patients treated with FOLFIRI were enrolled, revealed that the addition of bevacizumab significantly improved survival after first progression^13^. The ML18147 and BEBYP studies revealed the benefits of continuation of bevacizumab even after initial chemotherapeutic resistance^14,23^.

In China where people are mostly of Mongoloid decent, the evidence of RCT study in bevacizumab was not as sufficient as described above. The ARTIST study, a phase III RCT study, demonstrated that in Chinese population bevacizumab could increase mCRC survival significantly^24^. Oncologists in China mostly decide therapeutic regimens following international guidelines and additional experiences of bevacizumab administration are required. In the present study, we obtained similar results as RCT studies discussed above. The subtle differences in duration of survival and response rate may due to the different regimens and population.

The poor survival and the prognostic impact of *KRAS* mutation in the CRC have been previously reported^25^. It was shown that *KRAS* mutation status did not have a significant effect on the PFS in first-line treatment of mCRC^26^. Hurwitz *et al*. (2009) reported that the clinical benefit of bevacizumab in mCRC was independent of *KRAS* mutation status; bevacizumab provided significant clinical benefit in patients with mCRC expressing either mutant or wild-type *KRAS* ^27^. We explored the efficacy of bevacizumab-contained chemotherapy for stage IV CRC patients according to different *KRAS* mutation status. Our result revealed that patients with *KRAS* mutation had a shorter survival than patients with wild type *KRAS* wild type; however, the difference was not statistically significant.

Current evidence indicates that right- and left-sided CRC respond differently to treatment. Proximal and distal colon show different pathways to develop and tumorigenesis. The proximal colon originating from the embryonic midgut is perfused by the superior mesenteric artery, however the distal colon deriving from the hindgut is supplied by the inferior mesenteric artery^28^. *KRAS* mutations were more frequently found in the right than left Dukes’ C colon cancer^29^. The patients with right-sided CRC were older female, poorly differentiated and suffered from poorer survival^30^. The more active EGFR signaling in distal CRC meant that left-sided cancers benefited significantly more from cetuximab^31,32^. In addition, it was shown using data obtained from PROVETTA, AVF2107g and NO16966 trials, that even though efficacy of bevacizumab was independent of tumor location, patients with left-sided tumors had significantly better outcomes^33^.

In the current study, we analyzed the short and long term efficacy in different anatomic sites of mCRC treated with bevacizumab. We divided mCRC into right- and left-sided groups and found that tumor location was not a prognostic factor for survival. Although the median PFS, OS and response rate of right-sided mCRC were all worse than left-sided cancers, none of the differences achieved statistical significance, which is contradictory with two previous reports^33,34^. The difference is perhaps because of the different population and line of bevacizumab administration. This highlights the importance of similar studies in the context of different population and analytic setting.

### Statement of Ethics

All procedures performed in studies involving human participants were in accordance with the ethical standards of the institutional and/or national research committee and with the 1964 Helsinki declaration and its later amendments or comparable ethical standards.
